# Effects of Melatonin on Neurobehavior and Cognition in a Cerebral Palsy Model of *plppr5*−/− Mice

**DOI:** 10.3389/fendo.2021.598788

**Published:** 2021-02-22

**Authors:** Yuxiao Sun, Liya Ma, Meifang Jin, Yuqin Zheng, Dandan Wang, Hong Ni

**Affiliations:** Division of Brain Science, Institute of Pediatric Research, Children’s Hospital of Soochow University, Suzhou, China

**Keywords:** cerebral palsy, hypoxic-ischemic, *plppr5*, melatonin, neurobehavior

## Abstract

Cerebral palsy (CP), a group of clinical syndromes caused by non-progressive brain damage in the developing fetus or infant, is one of the most common causes of lifelong physical disability in children in most countries. At present, many researchers believe that perinatal cerebral hypoxic ischemic injury or inflammatory injury are the main causes of cerebral palsy. Previous studies including our works confirmed that melatonin has a protective effect against convulsive brain damage during development and that it affects the expression of various molecules involved in processes such as metabolism, plasticity and signaling in the brain. Integral membrane protein *plppr5* is a new member of the plasticity-related protein family, which is specifically expressed in brain and spinal cord, and induces filopodia formation as well as neurite growth. It is highly expressed in the brain, especially in areas of high plasticity, such as the hippocampus. The signals are slightly lower in the cortex, the cerebellum, and in striatum. Noteworthy, during development *plppr5* mRNA is expressed in the spinal cord, i.e., in neuron rich regions such as in medial motor nuclei, suggesting that *plppr5* plays an important role in the regulation of neurons. However, the existing literature only states that *plppr5* is involved in the occurrence and stability of dendritic spines, and research on its possible involvement in neonatal ischemic hypoxic encephalopathy has not been previously reported. We used *plppr5* knockout (*plppr5*−/−) mice and their wild-type littermates to establish a model of hypoxicischemic brain injury (HI) to further explore the effects of melatonin on brain injury and the role of *plppr5* in this treatment in an HI model, which mainly focuses on cognition, exercise, learning, and memory. All the tests were performed at 3–4 weeks after HI. As for melatonin treatment, which was performed 5 min after HI injury and followed by every 24h. In these experiments, we found that there was a significant interaction between genotype and treatment in novel object recognition tests, surface righting reflex tests and forelimb suspension reflex tests, which represent learning and memory, motor function and coordination, and the forelimb grip of the mice, respectively. However, a significant main effect of genotype and treatment on performance in all behavioral tests were observed. Specifically, wild-type mice with HI injury performed better than *plppr5*−/− mice, regardless of treatment with melatonin or vehicle. Moreover, treatment with melatonin could improve behavior in the tests for wild-type mice with HI injury, but not for *plppr5*−/− mice. This study showed that *plppr5* knockout aggravated HI damage and partially weakened the neuroprotection of melatonin in some aspects (such as novel object recognition test and partial nerve reflexes), which deserves further study.

## Introduction

Cerebral palsy (CP) is one of the most common causes of lifelong physical disability in children in most countries (1–3). Its prevalence in live births is approximately 2.0‰–3.5‰ (4, 5). However, cerebral palsy is not a disease entity in the traditional sense but is instead a group of clinical syndromes caused by nonprogressive brain damage in developing fetuses or infants. At present, many researchers believe that perinatal cerebral hypoxic ischemic (HI) injury or inflammatory injury are the main causes of cerebral palsy (1, 6–8). Because a complex of symptoms including motor dysfunction are caused by cerebral palsy, it seriously affects the quality of life of patients and applies great pressure to the family and society. Therefore, the study of cerebral palsy is particularly important. Some previous experiments showed that the long-term behavioral performance of the neonatal ischemic hypoxia animal model (HI) is consistent with the characteristics of cerebral palsy, and the model has been widely recognized (8).

Members of the *plppr* (*PRG*) family (*PRG1-PRG5*) can mediate the regeneration process and lysophosphatidic acid (LPA) activity in neurons and are known to participate in neuronal plasticity (9, 10). *Plppr4* drives cell autonomous signaling pathways to participate in the regulation of spinal density and subsequent memory formation (11, 12). Our previous research on haloperazine-induced recurrent seizures in rats found that *PRG-1* mRNA and protein levels are significantly upregulated in the hippocampus and cerebral cortex in response to neonatal convulsions, and these levels are maintained over the long term (13). *Plppr3* induces collateral branch growth in axons (14). *Plppr1* induces neurites that are resistant to growth inhibitors associated with brain injury and can help restore function after spinal cord injury (15). The *Plppr2* protein is highly expressed during the development and regeneration of synapses, can regulate synaptic lysophosphatidic acid (LPA) levels and is associated with epilepsy and brain damage.

*Plppr5* (*PRG5)* is a new member of the plasticity-related protein family. Integral membrane protein *plppr5* is specifically expressed in brain and spinal cord, and induces filopodia formation as well as neurite growth*. Plppr5* is highly expressed in the brain, especially in areas of high plasticity, such as the hippocampus. The signals are slightly lower in the cortex, the cerebellum, the stratum radiatum, and in striatum. Noteworthy, during development *plppr5* mRNA is expressed in the spinal cord, i.e., in neuron rich regions such as in medial motor nuclei (10, 16).

Autophagy inhibitor E64d pretreatment can improve convulsive brain injury in rats and downregulate the expression of *PRG-1, PRG-3, PRG-5*, cathepsin E and ApoE mRNA (17). Thomas Broggini suggested that *plppr5*, as an upstream molecule of ROCK, can overcome LPA and Nogo-A-induced neurite contraction caused by RhoA-ROCK-PIP5K kinase pathway activation (16). Recently, Tan et al. found that electroacupuncture stimulation may be involved in the neuroprotective effect of cerebral ischemia-reperfusion rats mediated through *plppr5/NogoA-LPA/RhoA* signaling (18).

Multiple studies have shown that the use of neuroprotective drugs can improve the prognosis of cases of cerebral palsy. For example, magnesium sulfate taken before premature delivery can significantly reduce the risk of cerebral palsy within 2 years of birth (19). Erythropoietin can limit inflammation and decrease the death of cells in many animal studies of hypoxic-ischemic injury (20, 21). Melatonin (n-acetyl-5-methoxytryptamine, Mel) is a multitask molecule, that plays a role both as a chronobiological hormone (hormone of darkness), and a mediator of immunological responses (22, 23). In addition to the pineal gland secreting melatonin, an increasing number of studies have shown that extra-pineal tissues secrete melatonin as well (24). Meanwhile, Melatonin is synthesized “on demand” in response to innate immune responses or certain inflammatory conditions (23). Because of its high fat solubility, ease of passing through the blood-brain barrier and cell membranes, and low toxicity, it has gradually become a promising neuroprotective drug (25). It is already in the preclinical trial stage in the treatment of convulsive brain injury (26, 27).

Naskar’s research showed that melatonin has been reported to restore lost striatal spines in the MPTP model of Parkinson’s disease (28); Chakraborty reported that melatonin protects against behavioral dysfunctions and dendritic spine damage in 3-nitropropionic acid-induced rat model of Huntington’s disease (29); Changes in dendritic complexity and synaptic plasticity are closely related to long-term neurological dysfunction after brain injury. In neurodevelopmental disorders and ischemic brain injury, a decrease in the number of dendritic branches and synaptic density can be found (30). So it is speculated that the two may have an internal connection. The neural plasticity signal pathway involved in *Plppr5* may be the target of the neuroprotective effect of melatonin, which is worthy of further study.

It is conceivable that the plasticity related protein family represented by *plppr5* may play a key role in recovering from brain injury. Neonatal hypoxic-ischemic encephalopathy is the most common brain injury in children, and moreover, neurodevelopment is very obvious during the development of the newborn. To the best of our knowledge, research on the role of *plppr5* in neonatal hypoxic ischemic encephalopathy has not yet been reported.

Therefore, in this study, we used *plppr5* gene knockout mice (*plppr*5−/−) and their wild-type littermates to establish a model of ischemic hypoxic brain injury (HI) to further explore the effects of melatonin on brain injury and the possible role of *plppr5* in melatonin’s protective effect against brain injury. These results will provide a stronger theoretical basis for the clinical use of melatonin in the treatment of ischemic hypoxic encephalopathy.

## Materials and Methods

### Animal Preparation

The generation of *plppr5* knockout mice was conducted in the GemPharmatech Co, Ltd (formerly known as Nanjing Biomedicine Research Institute of Nanjing University, Nanjing, China) using CRISPR/Cas9 technology, and gRNA was designed and transcribed *in vitro*:

gRNA1:5’-ACCACTAGGCAGTAGAGACT-3’,PAM : GGG;
gRNA2:5’-TGTGAGGACAATTGGCTCTA-3’,PAM : AGG;
gRNA3:5’-GGGCTTTGTCGTGGGTGGCG-3’,PAM : GGG;
gRNA4:5’-CACAGTCTCGTGGGAGGGCG-3’,PAM : GGG.

**Figure 1:**
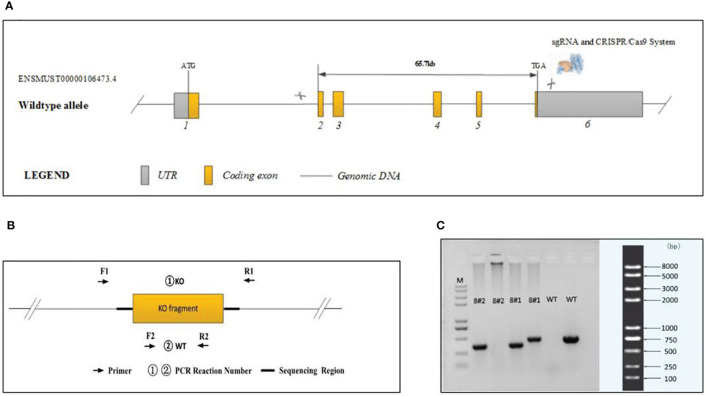
Generation of *plppr5*−/− mice and confirmation of *plppr5* deficiency at the DNA level. **(A)** Gene sequence insertion; **(B)** PCR analysis; **(C)** Samples 8#2 and 8#1 correspond to the *plppr*5−/− and *plppr*5+/- mice, respectively. M represents the DNA ladder.

Cas9 and gRNA were simultaneously injected into 200 fertilized mouse eggs. The Cas9 protein binds to the target site under the guidance of gRNA and causes DNA double-strand breaks, thereby achieving deletion of the base sequence of the target site. Final realization and gene knockout are shown in **Figure 1A**. A total of four positive F0 generation mice were obtained.

All procedures were reviewed and approved by the Institutional Animal Care and Use Committee of Soochow University (ethical code:SUDA20201020A01). We took adequate measures to minimize animal suffering, and the sample size is based on the sample size used in our group’s previous publications and other similar studies (31).

Experiments were performed on knockout mice backcrossed to C57BL/6 for over 15 generations at the time of experiment and several more generations in our own laboratory. The genotype of the transgenic mice was identified by PCR *via* genomic DNA prepared from tail biopsies using standard procedures. PCR Reaction Component was used as follows: 12.5μl of 2 × Taq Master Mix, Dye Plus, (Vazyme P112-03); 9.5μl of ddH_2_O;1μl of each primer (10pmol/µl); 1μl of Template (≈100ng/μl). And the following thermal cycling was used: 95 °C 5 min; followed by 20 cycles of 98 °C 30 s, 65 °C (-0.5 °C/cycle) 30 s, 72 °C 45 s; followed by 20 cycles of 98 °C 30 s, 55 °C 30 s,72 °C 45 s; 72 °C 5 min; 10 °C hold. The following primers were used to amplify the PCR fragments **Figures 1B, C**:

①KO : XM709934-*Plppr5*-KO-TF2 : 5’-AGCTGGTGTTACATTACAGGCAG-3’;
XM709934-*Plppr5*-KO-TR2 : 5’-GGGTTTCTTTCCACATAGTCACAG-3’.
②WT : XM709934-*Plppr5*-WT-TF1 : 5’-GTTCTCCAGTTCAATCATTGGG-3’;
XM709934-*Plppr5*-WT-TR1 : 5’-ATGCTGTATCCCGTGCTTTCTG-3’.

**Figure 2:**
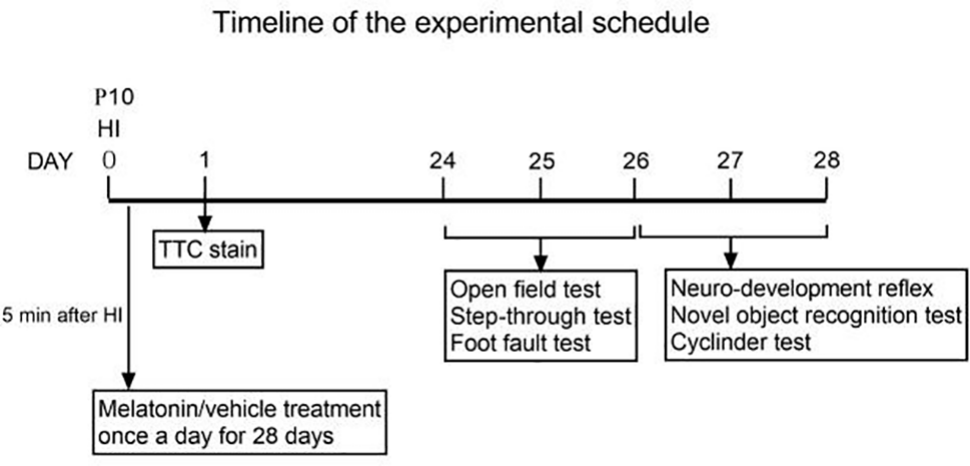
Timeline of the experimental schedule.

①KO primers were designed to generate PCR fragments of 414 bp for the KO allele and 67739 bp for the WT allele, the latter of which was not amplified due to its large size. To distinguish between the KO/WT and KO/KO mice, ②WT primers were designed to produce a 439 bp fragment for the WT allele only. A lot of experimental and clinical researches found that males show increase risk for brain-based developmental disorders including learning disabilities and cerebral palsy compared with females (32, 33). Therefore, in our experiment only male *plppr5*−/− mice and its wild-type littermates were used. We performed an experiment (**Figure 2**) during long-term follow-up.

### Group Allocation

The mice used for behavioral testing come from different litters, and at least 1 or 2 mice for each treatment group in every litter: KO+Veh, KO+Mel, WT+Veh, and WT +Mel. Mice were weaned 21 days after birth, then they were group housed. Every group was divided into two cages with 6 mice per cage. Another three cages of mice of each genotype with the same hypoxic ischemic brain injury and treatment were used for TTC staining. Furthermore, in order to identify the success of the animal model, we designated another group as sham for every genotype (KO represents *plppr5*−/− mice with HI injury, WT means wild-type mice with HI injury, Mel means melatonin, Veh means vehicle.).

### Animal Model of Hypoxia-Ischemia

The model of hypoxic-ischemic (HI) injury in newborn mouse pups was performed based on the well-established Rice Vannucci model (34). Briefly, C57/BL6 wild-type and plppr5 knockout postnatal day 10 (P10) pups (35) from the same parental generation were used. All of the pups were anesthetized with diethyl ether until they were fully anesthetized and unresponsive (36, 37). The left carotid artery was isolated, double ligated with a 5-0 surgical suture and cut between the ligations. Last, the skin was sutured back to close the incision. The entire procedure took no more than 6 min. The mice were allowed to recover for 1 h in their cages. The mice were then put in a 37°C chamber under a gas mixture of 8% oxygen and 92% nitrogen for 2 h. For the sham groups, sham animals received an incision but no ligation, and the pups were placed in a similar but not hypoxic environment. Finally, the pups were returned to their dams and kept in a standard environment. The operation was carried out in the afternoon for melatonin intervention would be in evening.

### TTC Staining

2,3,5-Triphenyltetrazolium chloride (TTC) is a redox indicator, which can be reduced by the mitochondrial enzyme succinate dehydrogenase to a fat-soluble compound. The normal brain tissue appears red, and the infarct areas are white (38). At 24 h after the HI insult, some pups were sacrificed, and the brains were extracted and then frozen at −20 °C for 10 min. Next, they were cut into ~2 mm sections coronally and then immersed in 1% TTC at 37 °C in a dark environment (10–20 min). Finally, the sections were immersed in a 4% paraformaldehyde solution overnight and photos were taken (39). Pictures of the stained slices were obtained and the infarct area was manually delineated using ImageJ. The degree of cerebral infarction is presented as the percentage of infarction volume to total brain volume.

### Melatonin Intervention

Melatonin was purchased from Sigma (St. Louis, MO, USA). Melatonin was administered after the hypoxic-ischemic insult in groups KO+Mel and WT+Mel. The dose was intraperitoneally injected daily according to body weight (0.1 mg/10 g body weight per day) at 5 min after the surgery and once every 24 h for 4 weeks (27). Meanwhile, the other two groups were injected with vehicle of the same volume (40) and at the same time in the evening (18:00–20:00). The concentration of melatonin was 2 mg/ml (27).

### Weight Monitoring

The body weight of the mice in each group was recorded every 4 days to estimate the physical development of the pups.

### Behavioral Evaluation of Adolescent Mice

Behavioral testing was performed in a quiet room with non-distracting, homogenous lighting. In order to explore the long-term effect of melatonin on behavior, we choose the time-point of 4 weeks after HI injury to take these behavioral testing. Specifically, open field test, step-through test and foot fault test were performed during 24-26 days after HI injury (PND 34-36). Neurodevelopment reflex tests, novel object recognition were all evaluated during 27-28 days after HI injury (PND 37-38). Testers are unclear about the experiment grouping and handling. The details are as follows:

#### Neuro-Developmental Reflex

Negative geotaxis reflex: the negative geotaxis reflex is considered to reveal the functions of vestibular and proprioception of mice. The mice were blindfolded and placed on a 45° inclined plywood surface with the head towards the ground. We recorded the time it took for the mouse to switch from a head-down to a head-up position. The shorter the time, the better the mouse’s reactivity was (41).

Cliff avoidance reflex: the cliff avoidance reflex is used to assess the ability of rodents to respond to adverse environments. The mouse was placed on the edge of a test bench with its forepaws and nose over the edge. We recorded the time from when the mouse turned away from the edge and completely reversed position. The shorter the time, the better the mouse’s reactivity was (42).

Forelimb suspension reflex: a metal rod (diameter of 0.5 cm) was fixed at a height of 50 cm from the ground, and the mice were suspended with their forepaws grasping it. The time the animal remained on the bar was recorded. The longer the time, the better the mouse’s reactivity was (43).

Surface righting reflex: each mouse was placed in a supine position and the time required to turn their body completely to the ground was recorded. Shorter times indicated faster reactions (41).

#### Novel Object Recognition Test

The novel object recognition test was performed in an open field test apparatus (40cm× 40cm× 30cm) under dim light in order to test the neurobiology of nonspatial memory in rodents. The entire test is divided into three stages: the first day is adaptation (T0) where the mouse is allowed to freely explore the open field for 5 min. Day 2 is training (T1), where the mouse is allowed to explore the arena with two identical triangular Lego toys and square Lego toys placed along the diagonal and glue them to the bottom of the box. The mice of each group were randomly placed in a test box with different Lego toys, and the mice were allowed to explore freely for 5 min. Testing (T2) takes place on Day 3: in the test phase after 24 h, one of the old objects is replaced with another shape object (that is, the same mouse faces a triangular toy and a square toy). Mice are born to like novelties; if the mice can recognize the familiar object, it will spend more time with the novel object. Object detection is defined as pointing the nose at the object from a distance of ≤ 2 cm, for instance, touching it with the forehead or nose to sniff or bite the object. The recognition index is used to indicate recognition memory, which is calculated as (Tnovel)/(Tnovel+Tfamiliar), as previously described (44).

#### Open Field Test

Mice were tested in the open field for general locomotor activity, anxiety, and willingness to explore. Animals were tested in an acrylic arena (72 × 72 × 50 cm). The floor of the arena was divided into 16 squares of 18 × 18 cm. To start each session, a mouse was placed in the center of the arena and allowed to explore for 5 min. After 24 h, each mouse was observed for 5 min. The arena was cleaned with 75% ethanol between trials to eliminate the smell left by the previous mouse. Observation indicators included time in the center area (defined as center duration time), the total grids traveled, rearing (defined as standing on the hindlimbs without touching the wall), and grooming of mice. The open score was calculated as (the total grids traveled + rearing) (45).

#### Step-Through Test (Passive Avoidance Response)

A DC pulse stimulus current was passed through the floor in a darkroom. The mice were first placed in a darkroom reaction box for 3 min, and the mice escaped to the bright room after the electric shock. At the beginning of the formal test, the mice were placed in the bright room with their backs facing away from the hole. The mice were shocked when they entered the dark room. The dark avoidance device automatically recorded the number of times the mice entered the dark room within 5 min, which is the number of errors and the time from when they first entered the dark room to avoid dark latency. We started the training experiment at 9:00 am on the first day and performed the test at 9:00 am the next day (24 h after the training experiment). We recorded the latency avoidance period and the number of errors of the mouse within 5 min (46, 47).

#### Foot Fault Test

The foot fault test was carried out to evaluate sensorimotor function after HI insult. The mice were allowed to walk on a grid, which is a homemade grid with a mesh size of 2 × 2 cm and a height of 50 cm above the ground. Before the test, every mouse was allowed to walk on it freely for 5 min, and 1 h later, the test was officially conducted. A wrong step was defined as limb fall or slip into the mires and the number of times the right forelimb and the right hindlimb were recorded separately (48).

#### Cylinder Test

The cylinder test was used to assess the asymmetry in the use of the forelimbs of the mice when subjected to ischemic damage. The mice were placed separately in a transparent cylinder (20 cm in diameter and 45 cm in height). The number of times the mice touched the wall of the cylinder over 5 min was used to calculate the asymmetry score of the mice after HI insult. The higher the score was, the more obvious the asymmetry of the limbs on both sides. Asymmetry of forelimb use and paw preference were calculated as (left (nonimpaired side) – right (impaired side))/(left + right) × 100% (49).

### Determination of Seizure Threshold

Survivors of neonatal HI often develop brain injury and neurologic disabilities (e.g., cognitive deficits and epilepsy) in later life. All of the groups are injected with penicillin (5.1 × 10^6^U/kg/d, i.p.) after behavioral evaluation. We recorded the time of the first seizure in the mouse and the seizure latency (min) (seizure threshold). The observation time was 90 min. According to the Racine classification, seizures are considered to occur if the seizure degree reaches level IV or above. Moreover, after the seizure started, the mouse was injected with 4% chloral hydrate immediately (50).

### Timm Staining

Timm staining is based on the staining of Zn^2+^-containing mossy fibers by a sulfide/silver stain as described previously. Briefly, the mice were deeply anesthetized and then fixed by transcardial perfusion with 0.9% NaCl followed by 0.3% Na_2_S in 100 mM phosphate buffer (PB) and 4% paraformaldehyde in 100 mM phosphate buffer (PB). After perfusion, the brains were postfixed in 4% paraformaldehyde overnight at 4°C. Coronal sections were cut at 30µm on cryostat and stained for mossy fibers using Timm staining. The processing solutions were obtained from Sinopharm Chemical Reagent Co., Ltd., China and were as follows: 50% Arabic gumc, 15ml Citric acid buffer (3.825g citric acid,3.525g sodium citrate),45ml 5.67% hydroquinone, and 0.5ml 17% silver nitrate (51–53).Then stained for 40–60 min at 37°C. After rinsing, the sections were dehydrated in alcohol, cleared in xylene, and mounted on slides with permount. Timm staining was analyzed at a magnification of 40 and 100 using an OLYMPUS PM20 automatic microscope (Olympus, Japan).

### Statistical Analysis

The data were analyzed using SPSS 17.0 statistical analysis software (SPSS, Chicago, IL, USA).Behavioral test analysis was based on *a priori* performance criteria, that is, the double-blind principle was adopted, and the data statisticians did not understand the grouping of experimental animals. Two-way factorial analysis of variance (ANOVA) and Bonferroni *post hoc* tests were used for the statistical analyses of the variable behavioral test and TCC staining of infarct volume. For the analysis of weight data, three-way ANOVA with repeated measures was used with Bonferroni *post hoc* tests. To ensure there was complete equality of the variances of the differences between all variations in the related groups, an assumption of sphericity was conducted with Mauchly’s test. If the assumption of sphericity was not met, the Greenhouse–Geisser correction was used. Normal distribution of the variables was assessed with the Shapiro-Wilk test for all variables in each treatment group. If the normal distribution was not satisfied, the data were transformed to a normal distribution. All data are presented as the mean ± standard deviation (SD). Throughout the study, *p values < 0.05 and **p values < 0.01 were considered significant and n.s., not significant. All graphs were created using GraphPad Prism version 8.0 software (GraphPad, San Diego, CA, USA).

## Results

### TTC Staining

**Figure 3:**
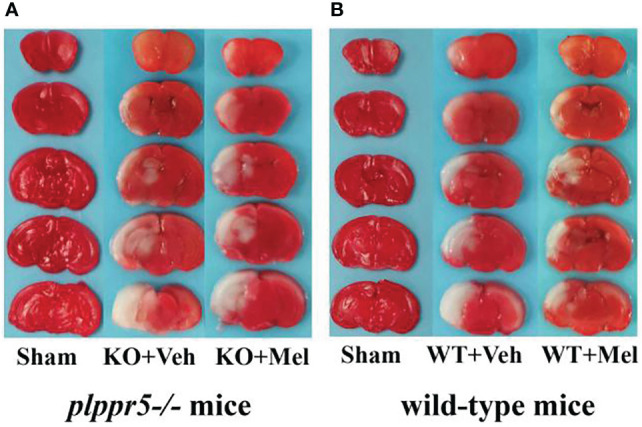
Photomicrographs of TTC-stained coronal slices showing brain infarction 24 h after HI: **(A)** *plppr5*−/− mice; **(B)** Wild-type mice. WT, wild-type mice with HI injury; KO, knockout mice with HI injury; Mel, melatonin; Veh, vehicle.

**Figure 4:**
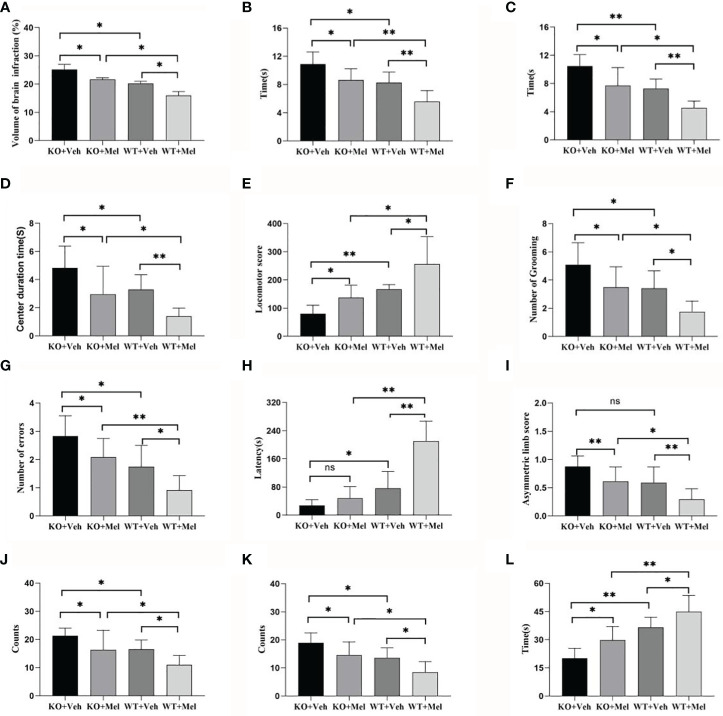
Behavioral test in wild-type mice with HI injury treated with vehicle or melatonin as well as knockout mice with HI injury receiving vehicle and melatonin treatments as indicated. **(A)** TTC staining; **(B)** Negative geotaxis reflex test; **(C)** Cliff avoidance reflex test; **(D)** Open field test-center duration time; **(E)** Open field test-locomotor score; **(F)** Open field test-number of grooming; **(G)** Step through test-number of errors; **(H)** Step through test-latency avoidance period; **(I)** Cylinder test; **(J)** Foot fault test-forelimb foot fault test; **(K)** Foot fault test-hindlimb foot fault test; **(L)** Seizure threshold. [Two-way ANOVA, Bonferroni *post hoc* test (details are shown in text)].

Infarct volume was used to evaluate brain damage at 24 h after HI injury. Three mice were subjected to TTC staining in each group. The results showed that for each genotype in the groups receiving melatonin or vehicle there was no significant interaction between genotype and treatment [F_(1,8)_=0.225, *P*=0.648], but there was a significant main effect of genotype [F_(1,8) _=55.280, *P* < 0.001] and treatment [F_(1,8)_ =29.354, *P* < 0.05] on infarct volume. Bonferroni *post hoc* testing revealed that the mice with melatonin-treatment had a decreased infarct volume relative to the vehicle-treated mice (*P*< 0.001) and that the wild-type mice with HI injury had a decreased volume relative to the knockout mice (*P*< 0.001) (**Table 1**, **Figures 3** and **4A**).

**Table 1:** Significant results for two-way analysis of variance.

		Treatment		Main Effect		Interaction Effect
		Veh (mean ± SD)	Mel (mean ± SD)	Genotype	Treatment	Genotype × Treatment
**TCC staining**						
Infarct volume	KO	0.252 ± 0.018	0.226 ± 0.006	F=55.28** p<0.001	F=29.354* P<0.05	F=0.225, P=0.648
	WT	0.202 ± 0.008	0.160 ± 0.013			
**Neurodevelopmental reflex**						
Negative geotaxis reflex test	KO	10.888 ± 1.711	8.623 ± 1.618	F=37.831** P<0.001	F=28.716** P<0.001	F=0.197, P=0.659
	WT	8.258 ± 1.504	5.583 ± 1.546			
Cliff avoidance reflex test	KO	10.476 ± 1.612	7.704 ± 2.541	F=41.204** P<0.001	F=30.337** P<0.001	F=0.004, P=0.950
	WT	7.251 ± 1.367	4.542 ± 0.979			
**Open field test**						
Center duration time	KO	4.829 ± 1.557	2.955 ± 2.000	F=13.280* P=0.001	F=23.474** P<0.001	F=0.305, P=0.583
	WT	3.292 ± 1.058	1.395 ± 0.586			
Locomotor score	KO	79.917 ± 30.330	137.417 ± 43.976	F=68.441** P<0.001	F=30.950** P<0.001	F=0.127, P=0.724
	WT	166.500 ± 16.839	256.583 ± 96.937			
Number of grooming	KO	5.083 ± 1.564	3.500 ± 1.446	F=21.084** P<0.001	F=19.078** P<0.001	F=0.013, P=0.911
	WT	3.417 ± 1.240	1.750 ± 0.754			
**Step-through test**						
Number of errors	KO	2.833 ± 0.718	2.083 ± 0.669	F=33.835** P<0.001	F=16.755** P<0.001	F=0.046, P=0.830
	WT	1.750 ± 0.754	0.917 ± 0.515			
Latency avoidance period	KO	27.423 ± 16.561	48.397 ± 32.748	F=57.605** P<0.001	F=23.363** P<0.001	F=2.865, P=0.098
	WT	76.167 ± 48.152	210.525 ± 56.346			
**Foot-fault test**						
Forelimb foot-fault test	KO	21.333 ± 2.708	16.333 ± 6.893	F=16.121** P<0.001	F=17.195** P<0.001	F=0.039, P=0.844
	WT	16.500 ± 3.289	11.000 ± 3.357			
Hindlimb foot-fault test	KO	18.917 ± 3.554	14.583 ± 4.719	F=25.385** P<0.001	F=17.270** P<0.001	F=0.110, P=0.742
	WT	13.583 ± 3.579	8.500 ± 3.729			
**Cylinder test**	KO	0.876 ± 0.189	0.613 ± 0.256	F=20.48** P<0.001	F=17.239** P<0.001	F=0.053, P=0.819
	WT	0.588 ± 0.282	0.295 ± 0.186			
**Determination of Seizure Threshold**	KO	20.078 ± 5.334	29.727 ± 7.286	F=52.722** P<0.001	F=17.117** P<0.001	F=0.078, P=0.782
	WT	36.552 ± 5.453	44.982 ± 8.624			

Bonferroni’s method is used for multiple comparisons with all possible pairwise differences of means. WT, Wild-type mice with HI injury; KO, knockout mice with HI injury; Mel, melatonin; Veh, vehicle; ns, Not Significant. *P < 0.05; **P < 0.001. [Two-way ANOVA, Bonferroni post hoc test (details are shown in text)].

### Weight Monitoring

**Figure 5:**
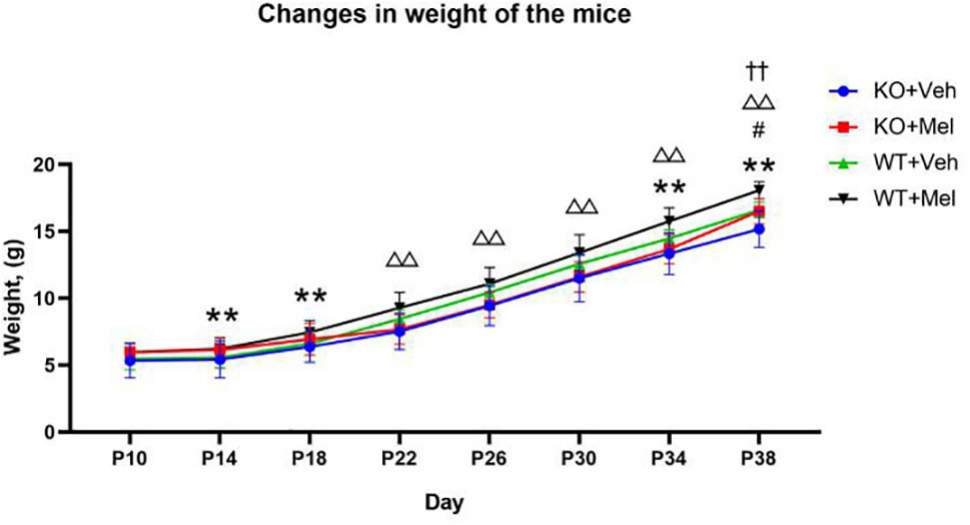
Changes in weight of the mice: **P < 0.001 for WT+Mel vs. WT; ^△△^P < 0.001 for KO+Mel vs. WT+Mel; ^#^P < 0.05 for KO+Mel vs. KO; ^††^P < 0.001 for KO vs. WT. [Three-way ANOVA, Bonferroni *post hoc* test (details are shown in text)].

Three-way ANOVA with repeated measures for each time and each genotype in all of the groups receiving the melatonin treatment or vehicle revealed no significant interaction between genotype, treatment and time [F_(1.562,1.242)_=1.258, *P*=0.305] on weight. A significant genotype × time [F_(2.299,25.288)_=17.135, *P*<0.001] and treatment × time [F_(2.438,26.820)_=3.811, *P*<0.05] interaction as well as a main effect of genotype [F_(1,11)_=7.709, *P*<0.05], treatment [F_(1,11)_=32.385, *P*<0.001], and time [F_(1,11)_=1671.417, *P*<0.001] were observed. Details are shown in **Table 2**. Bonferroni *post hoc* testing of the weight in each genotype group receiving melatonin treatment or vehicle indicated that all of the groups had a significant increase (*P*<0.05) in weight from P10 to P38 **(****Figure 5**). Bonferroni *post hoc* testing also revealed an increased weight in WT mice receiving vehicle (WT+Veh) at time P38 (*P*<0.05) compared to the KO mice treated with vehicle (KO+Veh). Meanwhile, WT mice receiving melatonin (WT+Mel) had increased weight at time P22 (*P*<0.05), P26 (*P*<0.05), P30 (*P*<0.05), P34 (*P*<0.05), and P38 (*P*<0.05) compared to the KO mice treated with melatonin (KO+Mel). Bonferroni *post hoc* testing also showed melatonin increased body weight compared to the vehicle at the times P14 (*P*<0.05), P18 (*P*<0.05), P34 (*P*<0.05), and P38 (*P*<0.001) in WT mice. Similarly, melatonin increased weight compared to the vehicle at the time P38 (*P*<0.05) in KO mice.

**Table 2:** Significant results for three-way analysis of variance.

	KO		WT	
	Veh(Mean ± SD)	Mel (Mean ± SD)	Veh (Mean ± SD)	Mel (mean ± SD)
P10	5.342 ± 1.281	5.993 ± 0.628	5.476 ± 0.839	5.973 ± 0.691
P14	5.422 ± 1.362	6.145 ± 0.855	5.576 ± 0.792	6.223 ± 0.844
P18	6.378 ± 1.149	6.941 ± 1.206	6.608 ± 0.849	7.45 ± 0.894
P22	7.526 ± 1.370	7.668 ± 1.108	8.463 ± 0.849	9.289 ± 1.151
P26	9.438 ± 1.479	9.515 ± 1.108	10.436 ± 0.611	11.091 ± 1.222
P30	11.516 ± 1.769	11.605 ± 1.126	12.583 ± 0.595	13.414 ± 1.341
P34	13.334 ± 1.554	13.673 ± 1.092	14.48 ± 0.652	15.763 ± 1.012
P38	15.178 ± 1.375	16.504 ± 0.938	16.615 ± 0.593	18.069 ± 1.012
genotype	F = 7.709*		P = 0.018	
treatment	F = 32.385**		P < 0.001	
time	F = 1671.417**		P < 0.001	
genotype × treatment	F = 0.610		P = 0.451	
genotype × time	F=17.135**		P < 0.001	
treatment × time	F=3.811*		P = 0.028	
genotype × treatment × time	F=1.258		P = 0.305	

Bonferroni’s method is used for multiple comparisons with all possible pairwise differences of means. WT, Wild-type mice with HI injury; KO, knockout mice with HI injury; Mel, melatonin; Veh, vehicle; ns, Not Significant ; *P < 0.05; **P < 0.001. [Three-way ANOVA, Bonferroni post hoc test (details are shown in text)].

### Neurodevelopmental Reflex

#### Negative Geotaxis Reflex and Cliff Avoidance Reflex Tests

Two-way ANOVA did not reveal significant genotype and treatment interaction effects on the performance in the negative geotaxis reflex test [F_(1,44)_=0.197, *P*=0.659] and cliff avoidance reflex test [F_(1,44)_=0.004, *P*=0.950]. However, a significant main effect of genotype [F_(1,44)_=37.831, *P*<0.001; F_(1,44)_=41.204, *P*<0.001] and treatment [F_(1,44)_=28.716, *P*<0.001; F_(1,44)_=30.337, *P*<0.001] was found for the negative geotaxis reflex test and cliff avoidance reflex test, respectively. Bonferroni *post hoc* testing of both behavioral tests revealed that the mice with melatonin-treatment performed better than the vehicle-treated mice, and the wild-type groups performed better than knockout groups (the *P*-values were all less than 0.001) (**Table 1**, **Figures 4B, C**).

#### Surface Righting Reflex Text

**Figure 6:**
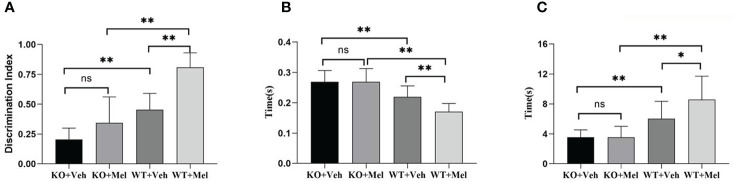
Behavioral test in wild-type mice with HI injury treated with vehicle or melatonin as well as knockout mice with HI injury receiving vehicle and melatonin treatments as indicated. **(A)** Novel object recognition test; **(B)** Surface righting reflex test; **(C)** Forelimb suspension reflex test. [Two-way ANOVA, Bonferroni *post hoc* test (details are shown in text)].

Two-way ANOVA for each genotype in all of the groups receiving the melatonin treatment or vehicle indicated a significant genotype × treatment interaction [F_(1,44)_=5.247, *P*<0.05] as well as a significant main effect of genotype [F_(1,44)_=49.422, *P*<0.001] and treatment [F_(1,44)_=5.247, *P*<0.05] on the surface righting reflex test. Bonferroni *post hoc* tests revealed no significant differences between the KO with HI injury group treated with melatonin (KO+Mel) and the vehicle group (KO+Veh) (*P>*0.05). However, a significant decrease in surface righting reflex time was observed in the wild-type with HI injury group treated with melatonin (WT+Mel) compared to the vehicle group (WT+Veh) (*P<*0.001). In addition, whether treated with melatonin or vehicle, wild-type mice with HI injury had a decreased time of surface righting reflex than the knockout mice [*P*<0.001, *P*<0.001, respectively] (**Table 3**, **Figure 6B**).

**Table 3:** Significant results for two-way analysis of variance.

		Treatment		Main Effect		Interaction Effect
		Veh (mean ± SD)	Mel (mean ± SD)	Genotype	Treatment	Genotype × Treatment
Forelimb suspension reflex test	KO	3.543 ± 0.987	3.558 ± 1.464	F=36.721** P<0.001	F=4.297* P=0.044	F=4.206*, P=0.046
	WT	6.021 ± 2.332	8.568 ± 3.125			
Surface righting reflex text	KO	0.269 ± 0.037	0.266 ± 0.043	F=49.422** P<0.001	F=5.247* P=0.027	F=5.247*, P=0.027
	WT	0.219 ± 0.037	0.171 ± 0.027			
Novel object recognition test	KO	0.205 ± 0.096	0.344 ± 0.217	F=67.604** P<0.001	F=32.52** P<0.001	F=6.204*, P=0.017
	WT	0.453 ± 0.138	0.808 ± 0.122			

WT, Wild-type mice with HI injury; KO, knockout mice with HI injury; Mel, melatonin; Veh, vehicle; *P < 0.05; **P < 0.001. [Two-way ANOVA, Bonferroni post hoc test (details are shown in text)].

#### Forelimb Suspension Reflex Test

Two-way ANOVA for each genotype in all of the groups receiving the melatonin treatment or vehicle indicated a significant genotype × treatment interaction [F_(1,44)_=4.206, *P*<0.05] as well as a significant main effect of genotype [F_(1,44)_=36.721, *P*<0.001] and treatment [F_(1,44)_=4.297, *P*<0.05] on the forelimb suspension reflex test. Bonferroni *post hoc* tests revealed no significant differences between the KO with HI injury group treated with melatonin (KO+Mel) and the vehicle group (KO+Veh) (*P*=0.978). However, a significant increase in forelimb suspension reflex time was observed in the wild-type with HI injury group treated with melatonin (WT+Mel) compared to the vehicle group (WT+Veh) (*P<*0.05). In addition, whether treated with melatonin or vehicle, wild-type mice with HI injury had an increased time of forelimb suspension reflex than knockout mice [*P*<0.001, *P*<0.001, respectively]. (**Table 3**, **Figure 6C**).

### Novel Object Recognition Test

Two-way ANOVA for each genotype in all of the groups receiving the melatonin treatment or vehicle indicated a significant genotype × treatment interaction [F_(1,44)_=6.204, *P*=0.017] as well as a significant main effect of genotype [F_(1,44)_=67.604, *P*<0.001] and treatment [F_(1,44)_=32.520, *P*<0.001] on the novel object recognition test. Bonferroni *post hoc* tests revealed no significant differences between knockout with HI injury group treated with melatonin (KO+Mel) and the vehicle group (KO+Veh) (*P*=0.054). However, a significant increase in novel object cognition index was observed in the wild-type with HI injury group treated with melatonin (WT+Mel) compared to the vehicle group (WT+Veh) (*P<*0.001). In addition, whether treated with melatonin or vehicle, wild-type mice with HI injury performed better than knockout mice [*P*<0.001, *P*<0.001, respectively] (**Table 3**, **Figure 6A**).

### Open Field Test

#### Center Duration Time

Two-way ANOVA showed no significant interaction between genotype and treatment [F_(1,44)_=0.305, *P*=0.583] for the center duration time. Main effects of genotype [F_(1,44)_=13.280, *P*=0.001] and treatment [F_(1,44)_=23.474, *P*<0.001] on the center duration time were found. Bonferroni *post hoc* testing of the center duration time revealed that the mice with melatonin treatment had a decreased center duration time than vehicle-treated mice (*P*< 0.001) and the wild-type mice with HI injury spent less time in the center than the knockout mice (*P*< 0.001) (**Table 1**, **Figure 4D**).

#### Locomotor Score

Two-way ANOVA showed no significant interaction between genotype and treatment [F_(1,44)_=0.127, *P*=0.724] for the locomotor score. Main effects of genotype [F_(1,44)_=68.441, *P*<0.001] and treatment [F_(1,44)_=30.950, *P*<0.001] were found on the locomotor score. Bonferroni *post hoc* testing of the locomotor score revealed that the mice with melatonin-treatment performed better than those who received vehicle-treatment (*P*< 0.001), and the wild-type groups performed better than the knockout groups (*P*< 0.001) (**Table 1**, **Figure 4E**).

#### Number of Grooming

Two-way ANOVA showed no significant interaction between genotype and treatment [F_(1,44)_=0.013, *P*=0.911] for the number of grooming. Main effects of genotype [F_(1,44)_=21.084, *P*<0.001] and treatment [F_(1,44)_=19.078, *P*<0.001] on the number of grooming were found. Bonferroni *post hoc* testing of the number of grooming revealed that the mice with melatonin-treatment had decreased numbers of grooming compared with the vehicle-treated mice (*P*< 0.001), and the wild-type groups had a decreased number of grooming compared with the knockout groups (*P*< 0.001) (**Table 1**, **Figure 4F**).

### Step-Through Test

#### Number of Errors

Two-way ANOVA showed no significant interaction between genotype and treatment [F_(1,44)_=0.046, *P*=0.830] for the number of errors. Main effects of genotype [F_(1,44)_=33.835, *P*<0.001] and treatment [F_(1,44)_=16.755, *P*<0.001] on the number of errors were found. Bonferroni *post hoc* testing on the number of errors revealed that the mice with melatonin-treatment had a decreased number of errors compared with the vehicle-treated mice (*P*< 0.001) and that the wild-type groups had a decreased number of errors compared with the knockout groups (*P*< 0.001) (**Table 1**, **Figure 4G**).

#### Latency Avoidance Period

Two-way ANOVA showed no significant interaction between genotype and treatment [F_(1,44)_=2.865, *P*=0.098] for the latency avoidance period. Main effects of genotype [F_(1,44)_=57.605, *P*<0.001] and treatment [F_(1,44)_=23.363, *P*<0.001] on the latency avoidance period were found. Bonferroni *post hoc* testing of the latency avoidance period revealed that the mice with melatonin-treatment had an extended latency avoidance period compared with the vehicle-treated mice (*P*< 0.001), and the wild-type groups had an extended latency avoidance period relative to the knockout groups (*P*< 0.001) (**Table 1**, **Figure 4H**).

### Foot Fault Test

Two-way ANOVA showed no significant interaction between genotype and treatment [F_(1,44)_=0.039, *P*=0.844] for the forelimb foot-fault test or for the hindlimbs [F_(1,44)_=0.110, *P*=0.742]. Main effects of genotype [F_(1,44)_=16.121, *P*<0.001] and treatment [F_(1,44)_=17.195, *P*<0.001] on the forelimb foot-fault test were found. Main effects of genotype [F_(1,44)_=25.385, *P*<0.001] and treatment [F_(1,44)_= 17.270, *P*<0.001] on the hindlimb foot-fault test were found as well. Bonferroni *post hoc* testing of the forelimb foot-fault test revealed that the mice with melatonin-treatment performed better than the vehicle-treated mice (*P*< 0.001), and the wild-type groups performed better than the knockout groups (*P*< 0.001). There was a statistically significant difference for the forelimb foot-fault test (*P*< 0.001) (**Table 1**, **Figures 4J, K**).

### Cylinder Test

Two-way ANOVA showed no significant interaction between genotype and treatment [F_(1,44)_=0.053, *P*=0.819] for the cylinder test. Main effects of genotype [F_(1_,_44)_=20.480, *P*<0.001] and treatment [F_(1_,_44)_=17.239, *P*<0.001] on the cylinder test were found. Bonferroni *post hoc* testing of the cylinder test revealed that the mice with melatonin-treatment performed better than the vehicle-treated mice (*P*< 0.001) and that the wild-type groups performed better than the knockout groups (*P*< 0.001) (**Table 1**, **Figure 4I**).

### Determination of Seizure Threshold

Two-way ANOVA showed no significant interaction between genotype and treatment [F_(1,44)_=0.078, *P=*0.782] for the determination of seizure threshold. Main effects of genotype [F_(1,44)_=52.722, *P*<0.001] and treatment [F_(1,44)_=17.117, *P*<0.001] on the determination of seizure threshold were found. Bonferroni *post hoc* testing of the determination of seizure threshold revealed that the mice with melatonin-treatment were higher than that of the vehicle-treated mice (*P*< 0.001) and the wild-type groups were higher than the knockout groups (*P*< 0.001) (**Table 1**, **Figure 4L**).

### Timm Staining

As can be seen from the results of Timm staining, the hippocampus in the four groups were all atrophy (all groups were given hypoxic-ischemic treatment), especially in the KO+Veh group, we can hardly see a clear hippocampus structure; However, hippocampal atrophy was alleviated when mice were treated with melatonin for 4 weeks especially in the WT+Mel and KO+Mel groups compared with the corresponding control groups (WT+Veh and KO+Veh). The results show that plppr5 knockout aggravated hippocampal atrophy caused by HI injury, while melatonin treatment significantly alleviate the atrophy.

In terms of hippocampal mossy fiber distribution and germination indicators, plppr5 knockout significantly inhibited the growth of mossy fiber, which was demonstrated in the comparison of KO+Veh and WT+Veh group without melatonin treatment, and in KO+Veh group, we can see rare and discontinuous moss fiber coloration only in CA3 but not DG (dentate gyrus) but no sprouting in both area. However, in WT+Veh group, moss fiber coloration can be seen in both CA3 and DG area, and it is more obvious in CA3.

**Figure 7:**
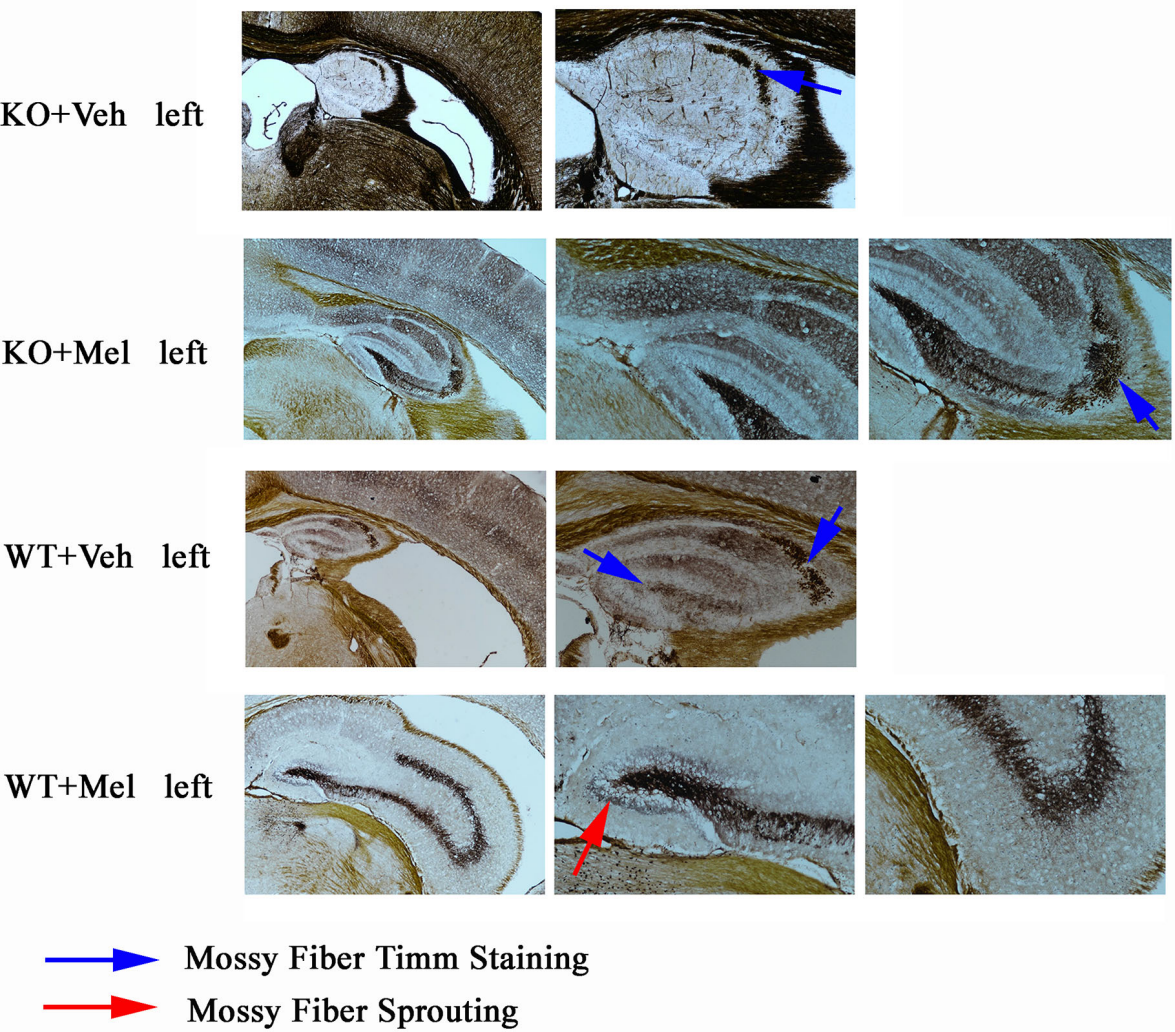
Timm staining results of left brain. Blue arrow means moss fiber staining. Red arrow means moss fire sprouting. Magnification is 40 times for first column (four pictures) and the others are 100 times.

The comparison of the two groups of KO+Mel and WT+Mel showed that the distribution of moss fibers in the DG and CA3 is clear. Meanwhile, there is no sprouting in the KO+Mel group, while the DG area of the WT+Mel group has obvious sprouting. Therefore, we can speculate that plppr5 knockout inhibited the axon regeneration of hippocampal granulosa cells (**Figure 7**).

## Discussion

The key findings of the current study are that *plppr*5 is related to the protective effect of melatonin, and the protective effect of melatonin on some neurobehaviors and cognition may be realized through the *plppr5* pathway. Meanwhile, the results show that *plppr5* knockout aggravated hippocampal atrophy caused by HI injury, while melatonin treatment significantly alleviate the atrophy.

Nerve development is unique to the early stage. It begins during embryogenesis and continues through fetal development and the neonatal period. Childhood and adolescence are important periods for learning, memory and emotional responses development. The establishment of memory and emotional responses is initiated in this period. Several risk factors, including perinatal hypoxic-ischemia (HI), can cause severe sequelae, such as chronic behavior disorders, social disability, impaired execution, and cerebral palsy in preterm infants (54). Clinical studies have confirmed that its effects on quality of life can persist through adulthood (55).

In this study, the behavioral changes in mice in the experiment simulated cerebral palsy in children, such as asymmetric limb use, gait changes, etc (49, 54). TTC staining after 24 h also confirmed unilateral carotid ligation causing lateral brain injury, which is consistent with previous studies (38, 39). We found that KO mice are more sensitive to ischemic injury than wild-type mice. Larger ischemic areas in the KO mice suggests that their neurons were more vulnerable to ischemia or abnormalities in blood flow, which needs further research. In general, *plppr5* knockout grow slower than wild-type mice with HI injury. This suggests that *plppr5* contributes to the growth and development of mice.

In most of the tests, there was no interaction between genotype and treatment, but a main effect of genotype and treatment on performance in the tests was found. What deserves our attention the most is that a significant interaction (genotype × treatment) was observed in the novel object recognition test, forelimb suspension test and the surface righting reflex test. Specifically, whether given melatonin or vehicle, mice in the WT group performed better than the KO group. Moreover, melatonin significantly reversed the poor performance in wild-type mice with HI injury but not in *plppr5*−/− mice with HI injury.

Only three of the above behavioral experiments detected interactions between genotype and treatment. The reason for these apparent discrepancies is not known; however, these tests assess different aspects of motor conditions. The novel object recognition test is a commonly used behavioral assay for the investigation of learning and memory, which relies solely on the inherent behavior of rodent searchers. Moreover, it appears to make use of a few brain regions and nervous conduction tracts. In addition, it is also commonly used to evaluate the effects of various drugs on brain damage (44).

The surface righting reflex test, which can be influenced by various factors, is a commonly used behavioral assay for motor function and coordination. As this test involves a reflex, there is no learning component, and it can be repeated throughout the experimental period. It has been reported that ischemia and hypoxia will delay the establishment of reflexes. Our experiment used P10 modeling, and testing was carried out 4 weeks after the modeling when the reflexes should have been established. The forelimb suspensiontest was used to evaluate motor function and deficits following neonatal HI. This suggests that with the current treatment regimen, melatonin improved the cognitive abilities of those mice that had relatively moderate and less severe injury and not those of mice with profound injury.

Numerous studies on the role of melatonin in various brain regions and signaling systems provide strong support for a neuroprotective role of melatonin. It was previously reported that melatonin activates protein kinase C and Rho-related kinases and induces neuritogenesis at early stages in N1E-115 cells and has the ability to regulate neuroplastic processes (56). Acute melatonin treatment increases apical dendritic length and dendritic complexity in the CA1 region (57). Bharati Sinha et al. (27) found that the expression of melatonin receptor 1 (MT1) in the brain of mice after HI injury was reduced. FJB staining showed that the neuron damage was significantly reduced, and the brain atrophy and hippocampal atrophy area were generally reduced. The OGD model further confirmed the role of the MT1 receptor. Silvia Carloni et al. (58) injected 15 mg/kg of melatonin 5 min after HI to study the effect of melatonin on cell necrosis and autophagy in the short term. Their experimental results showed that melatonin can block the downregulation of SIRT1 after HI. There was significantly reduced cell death at 1 h after HI, revealing the connection between SIRT1 and melatonin neuroprotection.

Function of melatonin is often triggered through interactions with its membrane receptors (MT1 and MT2) which are G protein-coupled receptors. MT1 and MT2 have been localized to discrete brain areas of the rodent nervous system, including the suprachiasmatic nucleus (SCN), cerebellum, thalamus, hippocampus, and peripheral tissues (59), for example, MT1 is expressed in the dentate gyrus, CA3, CA1 regions and the subiculum of the hippocampus (one of the major areas affected in newborn H-I injury) (27).

The hippocampus is an important part of the central nervous system (CNS) and is especially vulnerable to deleterious conditions, such as ischemia, epilepsy. Previous studies have elucidated the cognitive dysfunction-attenuating capabilities of exogenous melatonin administration which is consistent with our experimental results.

It is known that the cerebellum is critically involved in learning, balance, impulse control, memory and the formation of synapses. Luciana Pinato’s study showed that cerebellar can synthesize melatonin as a response to inflammation (23). Guissoni Campos, L. M. et al. reported that the effects of melatonin on cerebellum may be related to sensorimotor and neuroprotection (60). It should be mentioned that melatonin is a neuroprotective drug when cerebellar cells are attacked. However, if the normal balance of the NF-κB pathway is disturbed, melatonin can also cause cell death (61). LPS treatment resulted in the death of hippocampal and cortical neurons, but did not lead to the death of cerebellar neurons, indicating that the local production of melatonin can protect cerebellar neurons from LPS toxicity (23). In addition to its pivotal role in improving neurobehavioral deficits, Mel is able to scavenge free radicals, stimulate antioxidant defenses of the cells, and decrease the activity of pro-oxidant enzymes (62, 63).

Currently, most research indicates that *plppr5* is involved in the development and stabilization of dendritic spines (10). The body is a unified whole. Although some of our performance statistics indicated that there was no interaction between genotype and treatment, this does not mean that there was no interaction in molecular biology. Additional molecular mechanisms need further study. The *plppr5* gene is a member of the plasticity-related protein family and is strongly expressed during mouse brain development beginning on the 14th day of the embryonic period (E14), peaks at birth, and remains stable at least into early adulthood, but after brain injury, changes may occur. It affects synaptic morphology and changes the number of dendritic spines (16). *Plppr5* is involved in dendritic spine formation and stabilization. Dendritic spines are the postsynaptic components of most excitatory synapses. They are highly plastic during development, but are generally stable in adulthood. Studies have reported that their morphological or functional changes are closely related to the pathological state of the body, such as neurodegenerative diseases (10).

However, most dendritic spines in the adult cortex persist throughout life, the loss of dendritic spines after can detrimental to the functions of synaptic networks (64). That may explain that wild-type mice performed better than *plppr5* knockout mice in learning and memory.

In our experiment, melatonin was injected intraperitoneally 5 min after HI injury at 10 mg/kg and continued to be injected every 24 h for 4 weeks in order to observe the long-term effects of melatonin on cognition, learning and memory, motor ability, and neurodevelopmental reflexes. At present, the most commonly used method of drug dose conversion is body surface area-based (BSA-based) dose calculation and body weight based dose calculation. Food and Drug Administration has suggested that BSA-based dose calculation is the most appropriate method. However, when designing clinical trials, especially phase I and phase II, the dose, administration route, and timing are critical points to identify effective results. The safety, pharmacokinetics (PK), dosing, and effectiveness of melatonin in infants with HIE undergoing hypothermia is still unclear, which need further research.

What’s more, melatonin can regulate autophagy. A recent study provides evidence that autophagy can regulate synaptic plasticity at CA1 synapses and serves as a significant regulator of structural plasticity, synaptic strength, and memory consolidation (65). Autophagy activation was found only in the ischemic side of the brain after HI injury (66). Consistent with our experimental results, *plppr5* knockout weakens melatonin’s protection against learning and cognition. Thus, we suspect that melatonin might regulate autophagy through the plasticity-related molecules represented by *plppr5*, thereby achieving neuroprotective effects, which needs our further research to confirm.

To our knowledge, this is the first study designed to examine the interaction between *plppr5* and melatonin. A limitation of this study is that this is an one dose study (0.1 mg/10 g body weight per day, at 5 min after the surgery and once every 24 h for 4 weeks). In addition, *plppr5* knockout may have potential confounding effects on other tissues. (Neuron-specific promoters can be used to eliminate potential confusion, but our experiments did not.) Ma and colleagues’ research (67) showed that melatonin was detected in the urine of mice within 24 h after oral administration of melatonin, while the littermates may consume other mice’s urine while playing, which may affect their melatonin supplementation. However, due to the need to consider the sex, genotype and different treatment groups in the same litter, the influence of this factor cannot be excluded before weaning, and dividing the cage immediately after weaning can minimize its impact. This is indeed a question worth exploring, and relevant experiments can be specially designed to explore.

To summarize, melatonin was administered at a certain dose immediately after HI, which had a protective effect on the mice and could reduce later injury; at the same time, the HI injury was aggravated when *plppr5* was knocked out. These data suggest that the *plppr5* gene plays a protective role in HI injury, and it is related to the protective role of melatonin. Our results provide new insights into reducing the delayed neurological damage caused by hypoxia ischemia. Further research is needed in regard to the forelimb suspension reflex, surface righting reflex and novel object recognition test, especially the underlying molecular mechanisms of these abnormalities.

## Data Availability Statement

The original contributions presented in the study are included in the article/supplementary material. Further inquiries can be directed to the corresponding author.

## Ethics Statement

The animal study was reviewed and approved by Institutional Animal Care and Use Committee of Soochow University.

## Author Contributions

HN designed the study. YS and HN analyzed the data and wrote the manuscript. YS, LM, YZ, MJ, and DW were the operators of the experiment and were responsible for the statistical analysis of data. All authors contributed to the article and approved the submitted version.

## Funding

This work was supported by the National Natural Science Foundation of China (81871024 and 81471337), the key talent’s subsidy project in science and education of Department of Public Health of Jiangsu Province (ZDRCC2016008), and the Postgraduate Research & Practice Innovation Program of Jiangsu Province (KYCX20 2722).

## Conflict of Interest

The authors declare that the research was conducted in the absence of any commercial or financial relationships that could be construed as a potential conflict of interest.
