# Corrigendum: Effects of Melatonin on Neurobehavior and Cognition in a Cerebral Palsy Model of *plppr5−/−* Mice

**DOI:** 10.3389/fendo.2022.879685

**Published:** 2022-03-14

**Authors:** Yuxiao Sun, Liya Ma, Meifang Jin, Yuqin Zheng, Dandan Wang, Hong Ni

**Affiliations:** Division of Brain Science, Institute of Pediatric Research, Children’s Hospital of Soochow University, Suzhou, China

**Keywords:** cerebral palsy, hypoxic-ischemic, *plppr5*, melatonin, neurobehavior

In the article as published originally, the incorrect nomenclature was used when referring to several members of the *plppr* family in the second paragraph of the *Introduction*.

The sentence “*Plppr1* drives cell autonomous signaling pathways to participate in the regulation of spinal density and subsequent memory formation (11, 12)” should read “*Plppr4* drives cell autonomous signaling pathways to participate in the regulation of spinal density and subsequent memory formation (11, 12)”.

The sentence “*Plppr2* induces collateral branch growth in axons (14)” should read “*Plppr3* induces collateral branch growth in axons (14)”.

The sentence “*Plppr3* induces neurites that are resistant to growth inhibitors associated with brain injury and can help restore function after spinal cord injury (15)” should read “*Plppr1* induces neurites that are resistant to growth inhibitors associated with brain injury and can help restore function after spinal cord injury (15)”.

The sentence “The *Plppr4* protein is highly expressed during the development and regeneration of synapses, can regulate synaptic lysophosphatidic acid (LPA) levels and is associated with epilepsy and brain damage.” should read “The *Plppr2* protein is highly expressed during the development and regeneration of synapses, can regulate synaptic lysophosphatidic acid (LPA) levels and is associated with epilepsy and brain damage.”

The authors apologize for this error and state that this does not change the scientific conclusions of the article in any way. The original article has been updated.

## Publisher’s Note

All claims expressed in this article are solely those of the authors and do not necessarily represent those of their affiliated organizations, or those of the publisher, the editors and the reviewers. Any product that may be evaluated in this article, or claim that may be made by its manufacturer, is not guaranteed or endorsed by the publisher.

